# Forskolin increases the effect of everolimus on aromatase inhibitor-resistant breast cancer cells

**DOI:** 10.18632/oncotarget.25217

**Published:** 2018-05-04

**Authors:** Takanori Hayashi, Masahiro Hikichi, Jun Yukitake, Toru Wakatsuki, Eiji Nishio, Toshiaki Utsumi, Nobuhiro Harada

**Affiliations:** ^1^ Department of Biochemistry, School of Medicine, Fujita Health University, Aichi, Japan; ^2^ Department of Breast Surgery, School of Medicine, Fujita Health University, Aichi, Japan; ^3^ Department of Clinical Immunology, School of Health Sciences, Fujita Health University, Aichi, Japan; ^4^ Department of Health Science, School of Medicine, Fujita Health University, Aichi, Japan; ^5^ Department of Obstetrics and Gynecology, School of Medicine, Fujita Health University, Aichi, Japan

**Keywords:** everolimus, aromatase inhibitors resistance, long-term estrogen-deprived cells, PP2A, breast cancer

## Abstract

Aromatase inhibitor (AI) resistance is a major obstacle in the treatment of estrogen receptor-positive breast cancer. Everolimus (EVE) ameliorates AI-resistant breast cancer and is therefore used in cancer treatment. However, some patients show resistance to EVE. Here, we used 30 clones of long-term estrogen-deprived (LTED) MCF-7 cells as a model of AI-resistant breast cancer. We examined changes in protein phosphatase type 2A (PP2A) and cancerous inhibitor of PP2A (CIP2A), a negative regulator of PP2A, in LTED cells treated with EVE. In LTED cells with high sensitivity to EVE, CIP2A expression decreased at low EVE concentrations; however, in LTED cells poorly sensitive to EVE, CIP2A and PP2A did not change upon exposure to EVE. Therefore, we hypothesized that there is a relation between expression of CIP2A and sensitivity to EVE. Knockdown of CIP2A increased the sensitivity to EVE in three clones poorly sensitive to EVE. Additionally, we found that treatment with FSK, which activates PP2A, increased the sensitivity of the cells to EVE. Our data point to CIP2A and PP2A as novel therapeutic targets for AI-resistant breast cancer.

## INTRODUCTION

Estrogen plays a crucial role in the development and progression of estrogen receptor alpha (ERα)-positive breast cancer [1, 2]. Aromatase is a key enzyme in estrogen synthesis, and the use of aromatase inhibitors (AIs), such as letrozole, anastrozole and exemestane, in the adjuvant setting is regarded as a standard approach in postmenopausal women with ERα-positive breast cancer [3–5]. However, some breast cancer patients develop resistance to AIs following treatment [6]. One of the mechanisms causing AI resistance is the aberrant activation of ERα, dependent on its phosphorylation on serine 167 (S167) through the phosphatidylinositol 3-kinase (PI3K)-Akt-mammalian target of rapamycin (mTOR) signaling pathway [7–9], which regulates several cellular functions including cell growth, survival, and apoptosis [10, 11]. Inhibition of the PI3K-Akt-mTOR signaling pathway is expected to improve AI resistance. Specifically, some studies have indicated that treatment with everolimus (EVE), an mTOR inhibitor, in combination with exemestane, is associated with a 6-month improvement in women with resistance to non-steroidal AIs [12, 13]. Therefore, the U.S. Food and Drug Administration (FDA) has approved EVE for the treatment of advanced-stage, ERα-positive, human epidermal growth factor receptor 2 (HER2)-negative breast cancer in postmenopausal women that have already been treated with letrozole or anastrozole [12, 14–16].

The protein phosphatase type 2A (PP2A), a widely conserved protein serine/threonine phosphatase, is a key tumor suppressor that regulates the PI3K-Akt signaling pathway and has high relevance in human cancer [17–19]. Our previous report suggested that the inhibition of PP2A increases phosphorylation of ERα on S167 and estradiol (E_2_)-independent MCF-7 cell proliferation [20]. PP2A is regulated by cancerous inhibitor of PP2A (CIP2A) [21, 22]. CIP2A was initially identified as a tumor-associated autoantigen in gastric and liver cancer [21] and is involved in therapy resistance [23–25]. CIP2A also plays a role in breast cancer. A report indicates that estrogen controls the expression of CIP2A via the epidermal growth factor receptor [26]. Additionally, Yu *et al.* have investigated the expression and the regulatory effects of CIP2A in breast cancer and the correlation between CIP2A expression and prognosis of breast cancer [27]. A study has shown that CIP2A augments cell proliferation via the Akt signaling pathway [28]. A different report has indicated that genistein, a phytoestrogen, downregulates CIP2A, and has associated its intake with reduced breast cancer risk [29]. In this study, we investigated if CIP2A plays a role in the acquisition of resistance to estrogen depletion, which occurs when AI resistance develops.

Our previous study has shown that inhibition of PP2A increased ER phosphorylation and induced resistance to estrogen depletion and long term estrogen deprived (LTED) cells show lower levels of PP2A and Akt activation compared to MCF-7 [30]. Therefore, estrogen depletion might cause Akt signaling activation. Akt activation leads to cell proliferation and ER phosphorylation, which is one of the causes of anti-estrogens resistance. In this study, we show that okadaic acid (OA) and calyculin A (CalA), two PP2A inhibitors, induce resistance of the cells to estrogen depletion.

MCF-7 are defective in double-strand break repair (DSBR) and DSBR genes are involved in MCF-7 genomic instability [31]. Therefore, we decided to investigate the effect of long term estrogen deprivation (a model of AI resistance [32–34]) in single-cell clones. We established 30 clones of long-term estrogen-deprived (LTED) cells and investigated their sensitivity to EVE and their expression of PP2A and CIP2A upon EVE treatment. We found that EVE strongly activates Akt in cells resistant to EVE and strong activation of Akt may be responsible for the drug resistance. Additionally, we found that forskolin (FSK), a PP2A activator [35], inhibits cell growth in LTED cells. We also investigated whether the treatment with FSK affected the EVE sensitivity of the LTED clones.

## RESULTS

### PP2A inhibitors induce AI resistance in E_2_-deprived MCF-7

**Figure 1:**
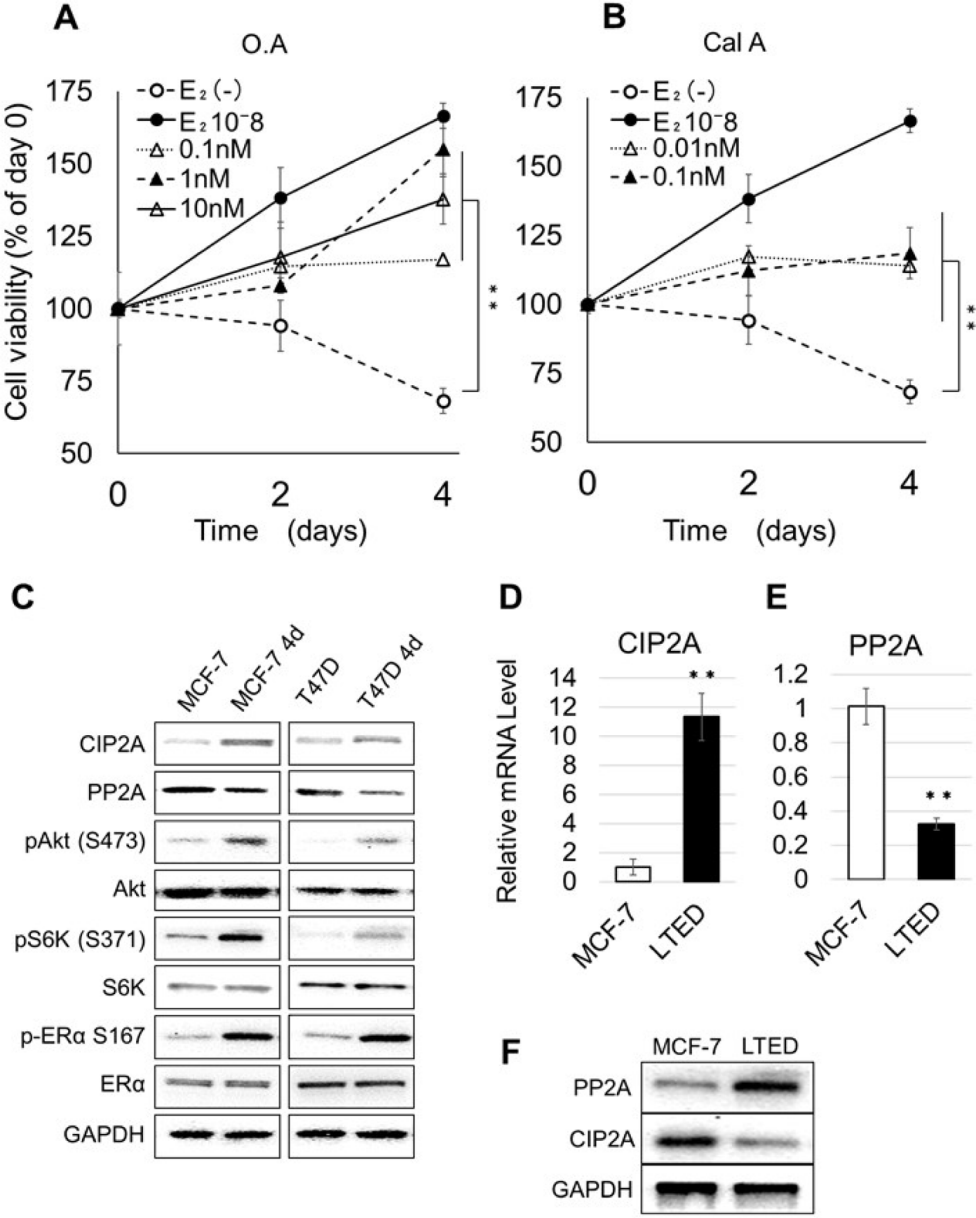
Effect of PP2A inhibition on the proliferation of E_2_-depleted cells (**A–B**) MCF-7 cells were cultured in the presence of estrogen-free medium or medium supplemented with E_2_, OA (A), or CalA (B) at the indicated concentrations. The number of cells at day 0 was considered as 100%. Cells were cultured for 2 or 4 days. The number of cells at each time point was calculated as percent of the number of cells at day 0. Data represent the mean ± SEM (*n* = 3 per treatment group. ^*^P < 0.05; ^**^P < 0.01). (**C**) MCF-7 and T47D were cultured for 4 days in medium without or with E_2_; total lysates were extracted and analyzed by western blotting. The image represents cropped areas of the PVDF membrane, each area indicating the reactivity of the indicated antibody. GAPDH was used as a loading control. (**D–E**) Relative mRNA levels of PP2A and CIP2A in MCF-7 cultured with E_2_ and LTED. Data represent the mean ± SEM (*n* = 3 per treatment group). (**F**) Western blot analysis of the expression of PP2A and CIP2A in MCF-7 and LTED.

The estrogen responsive breast cancer cells MCF-7 grow steadily in medium supplemented with E_2_ (10^–8^ M, Figure 1A). When cultured for 4 days in medium without E_2_, their number was greatly reduced (32% reduction, compared to cells at day 0). However, when MCF7 were cultivated for 4 days without E_2_ and in the presence of increasing concentrations of the PP2A inhibitor OA (0.1, 1, or 10 nM) their number increased compared to control cells (grown in medium without OA). Similar, though less pronounced, results were obtained when the PP2A inhibitor CalA was added to the cells instead of OA (concentrations of 0.01, 0.1 or 1 nM, Figure 1B). Cells incubated with 1 nM CalA died because of CalA cytotoxicity: therefore, the corresponding data are not shown. We have already reported that PP2A inhibition markedly increases the levels of ER phosphorylated on S167 (pER [S167]) [20]. The data obtained here confirm that E_2_-dependent cell proliferation and PP2A are closely related.

Next, we investigated the effect of E_2_ deprivation on the activation of the Akt pathway in MCF-7 and T47D cells; we chose to analyze also T47D in this experiment to verify if E_2_ depletion had the same effect in two different ER-positive cell lines. MCF-7 and T47D were cultured for 4 days in medium with or without E_2,_ and cell lysates were extracted and subjected to western blotting, using glyceraldehyde 3-phosphate dehydrogenase (GAPDH) as loading control (Figure 1C). In cells grown without E_2_ (MCF-7 4d and T47D 4d) we detected increased levels of CIP2A, Akt phosphorylated on S473 (pAkt [S473]), S6 kinase phosphorylated on S371 (pS6K [S371]) and pER (S167) and decreased levels of PP2A compared to cells grown in medium containing E_2._

We have previously shown that that LTED cells have lower levels of PP2A compared with MCF-7 [30]. Here, we confirmed these data for CIP2A and PP2A, at the mRNA (Figure 1D and 1E) and protein (Figure 1F) levels. These data suggest that the abnormal activation of the Akt signaling pathway results from changes in PP2A and CIP2A.

### Establishment of 30 clones of an AI-resistant breast cancer cell model (LTED cells) and analysis of their susceptibility to EVE

**Figure 2:**
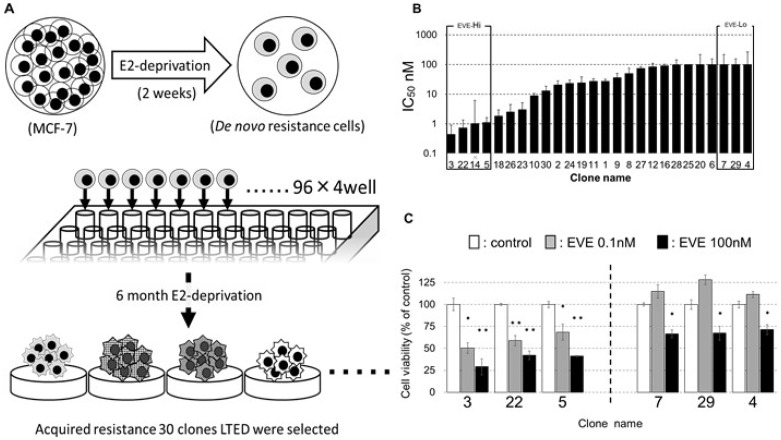
Establishment of the LTED clones (**A**) When MCF-7 cells were cultured in E_2_-depleted medium for two weeks, the cell number decreased, and cell division stopped. Arrested MCF-7 were seeded in 96-well plates with the limiting dilution method and cultured in E_2_-free medium. The resulting clones were named LTED clones, numbered in the order in which proliferation was confirmed and used for experiments. We established 30 clones in 6 months. (**B**) MCF-7 cells were incubated without or with EVE (0.01–100 nM) for 4 days. The medium was changed every 2 days. The cell number was measured with a CCK-8 kit. The relative cell numbers are given as values relative to cell number without EVE treatment. The concentration of EVE associated with the number of cells equal to 50% of their number at day 0 indicated the IC50. (**C**) Viability of cells differentially sensitive to EVE when exposed to 0.1 or 100 nM EVE; 3, 22 and 5 are clones highly sensitive to EVE; 7, 29 and 4 are clones poorly sensitive to EVE (*n* = 3 per treatment group).

EVE is an agent that increases the susceptibility to AIs in patients with breast cancer: it decreases the phosphorylation levels of pER (S167) and is used to limit the E_2_-independent proliferation of breast cancer cells (AI-resistant breast cancers). We hypothesized that the sensitivity to EVE and the expression of PP2A and CIP2A were closely related and decided to investigate this effect in cell clones. This endeavor required the creation of several clones: several studies indicate indeed that, to investigate the mechanism of drug resistance it is necessary to compare a large number of cell lines [36–39]. The process through which we generated the LTED cells partially differs from similar procedures in the literature [32–34]. In brief, MCF-7 cells were cultured for 2 weeks in medium without E_2_ (to simulate the clinical response to AIs therapy). From these cells, 30 LTED clones (resembling breast cancer cells resistant to AIs) were obtained with the limiting dilution method, cultivating single cells in 96-well plates for six months in medium without E_2_ (Figure 2A). The clones were incubated with various concentrations of EVE (between 0.01 and 100 nM) for 4 days, and the concentration of the drug at which the number of the cells was 50% of their number at day 0 was measured and defined as IC50 (Supplementary Figure 1). We selected three clones highly responsive to EVE, numbers (nos.) 3, 22 and 5 (indicated as EVE-Hi) and three poorly responsive to EVE, nos. 7, 29 and 4 (indicated as EVE-Lo, Figure 2B). Clone no. 14 was excluded from this study, because its growth rate was remarkably slow. EVE-Hi and EVE-Lo cells were incubated with or without EVE (0.01 or 100 nM) for 4 days. The number of EVE-Hi cells decreased significantly when cells were exposed to 100 nM EVE. The decrease in viability was less pronounced when the cells were grown with 0.01 nM EVE. Contrarily, the number of EVE-Lo cells increased when the cells were exposed to 0.01 nM EVE and decreased when cells were grown with 100 nM EVE; in the latter case, however, the decrease in viability was less pronounced than that in the EVE-Hi cells grown at the same concentration of EVE (Figure 2C).

### EVE decreases CIP2A in EVE-Hi cells

**Figure 3:**
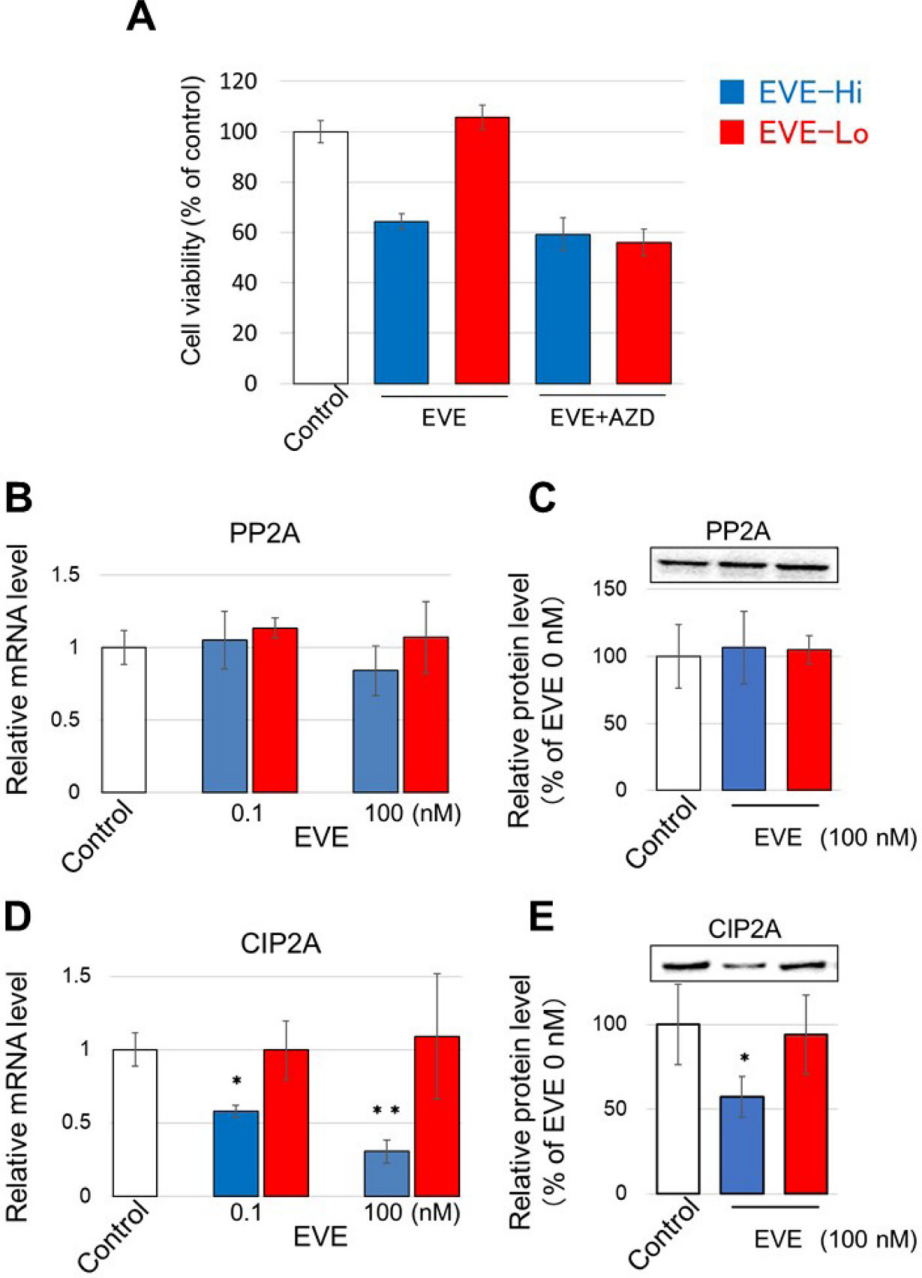
Effect of EVE on PP2A and CIP2A expression (**A**) EVE-Hi clones (nos. 3, 5 and 22, blue bars) and EVE-Lo clones (nos. 4, 7 and 29, red bars), were incubated with 0.1 nM EVE and 100 nM AZD. The medium was changed every 2 days. The number of the cells was measured with a CCK-8 kit. (**B–E**) The relative mRNA and protein levels of PP2A (A and C, respectively) and CIP2A (B and D, respectively) were evaluated in EVE-Hi and EVE-Lo clones treated with EVE at the concentrations indicated. GAPDH was used for normalization (*n* = 3 per treatment group ^*^P < 0.05; ^**^P < 0.01).

EVE inhibits mTOR and activates Akt in breast cancer cells [40]. The important regulators of Akt, PP2A and CIP2A are involved in the resistance of the cells to estrogen depletion [20, 30]. Therefore, we hypothesized that PP2A and CIP2A may play a role in the effect of EVE on cell growth. First, we investigated the relation between EVE sensitivity and the Akt signaling in general. We found that the difference in sensitivity to EVE between EVE-Hi and –Lo cells disappeared when cells were co-treated with the Akt inhibitor AZD5363 (AZD, Figure 3A). Next, we investigated if EVE sensitivity and PP2A and CIP2A expression were correlated. For this purpose, we analyzed the levels of the two proteins in EVE-Hi and -Lo cells. We did not find any significant difference in the levels of PP2A when the cells were incubated in the presence of 100 nM EVE for 24 h (Figure 3B and 3C). Contrarily, we found a significant decrease in CIP2A mRNA and protein in the EVE-Hi cells incubated with 100 nM EVE (Figure 3D and 3E). Therefore, we concluded that EVE interferes with the expression of CIP2A.

### Knockdown of CIP2A inhibits EVE-Lo cells proliferation

**Figure 4:**
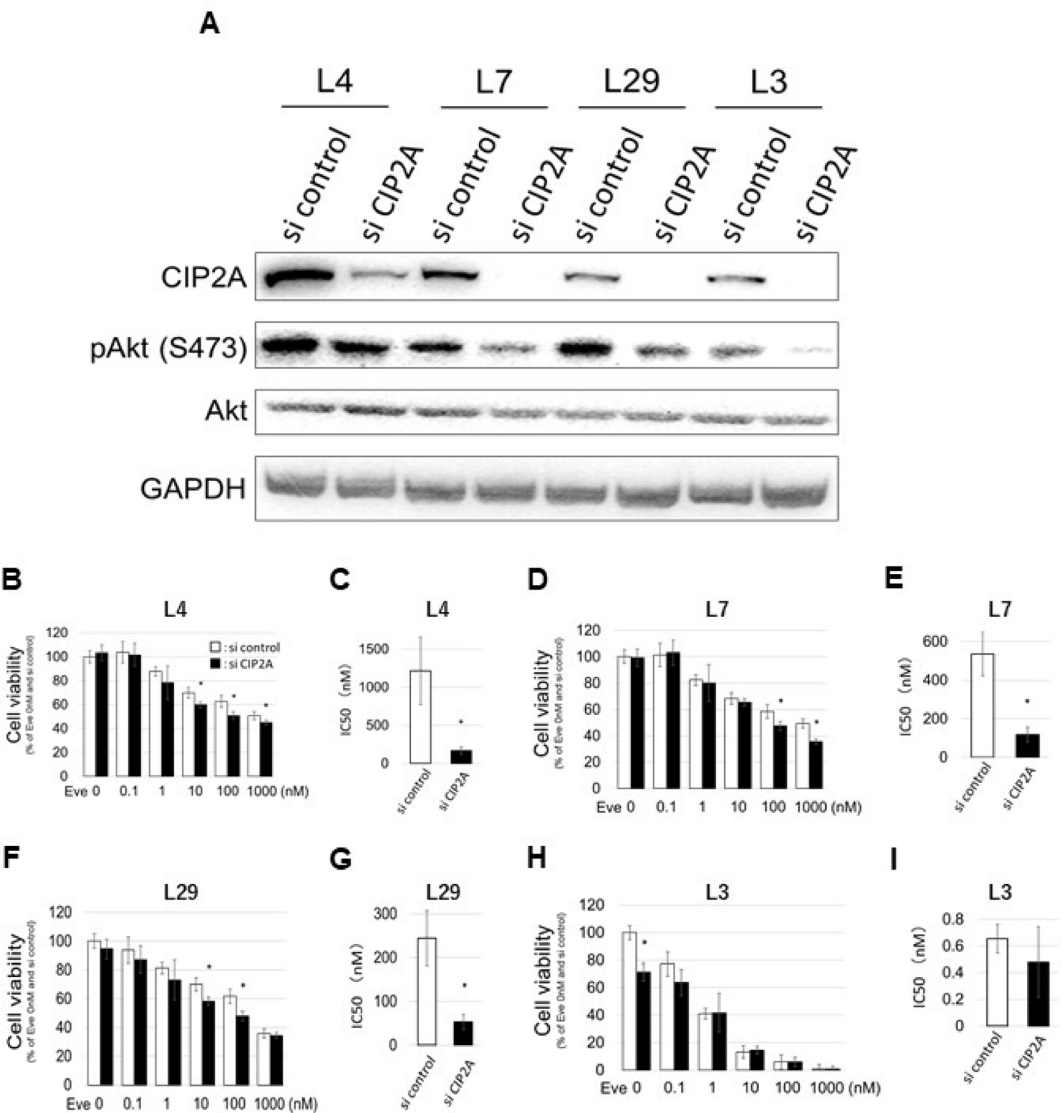
Effect of CIP2A gene knockdown on EVE sensitivity (**A–I**) Cells were transfected with CIP2A siRNA 24 h before EVE treatment (0–1000 nM) and used in different assays. (A) Western blot analysis of the expression of CIP 2A, pAkt (S473) and Akt. The image represents cropped areas of the PVDF membrane, each area indicating the reactivity of the indicated antibody. GAPDH was used as a loading control. (**B–I**) Cell viability and IC50 (concentration of EVE associated with 50% reduction in cell number) calculation in EVE-Lo clones no. 4 (L4; B, C), 7 (L7; D, E), 29 (L29; F, G), and EVE-Hi clone no. 3 (L3; H, I) (*n* = 3 per treatment group. ^*^P < 0.05).

To confirm whether CIP2A plays a role in the reduced cell viability induced by EVE, we investigated the effect of EVE on the cells in which the expression of CIP2A had been attenuated by RNA interference. First, we confirmed the reduction of CIP2A and pAkt (S473) upon CIP2A knockdown in all clones (Figure 4A). Three EVE-Lo clones (nos. 4, 7 and 29) and the EVE-Hi clone no. 3 were treated with various amounts of EVE (between 0.1 and 1000 nM) for 4 days and the number of cells was measured with a cell counting kit 8 (CCK-8). Upon CIP2A knockdown, we found a significant difference in the viability of cells of clone no. 7 grown with EVE at concentrations of 10, 100 and 1000 nM, compared with control cells (si control, treated with control small interfering RNAs, Figure 4B). This effect was more noticeable in clones nos. 4 and 29 (Figure 4D and 4F). On the other hand, cells of clone no. 3 showed a significant variation in viability upon CIP2A interference only when the cells were grown in the absence of EVE (Figure 4H). In the EVE-Lo cells, a significant decrease in IC50 was confirmed for all clones upon CIP2A knockdown (Figure 4C, 4E and 4G). The same treatment, however, was not associated to a change in EVE IC50 in clone no. 3 (Figure 4I).

### PP2A activation by forskolin decreases EVE resistance

**Figure 5:**
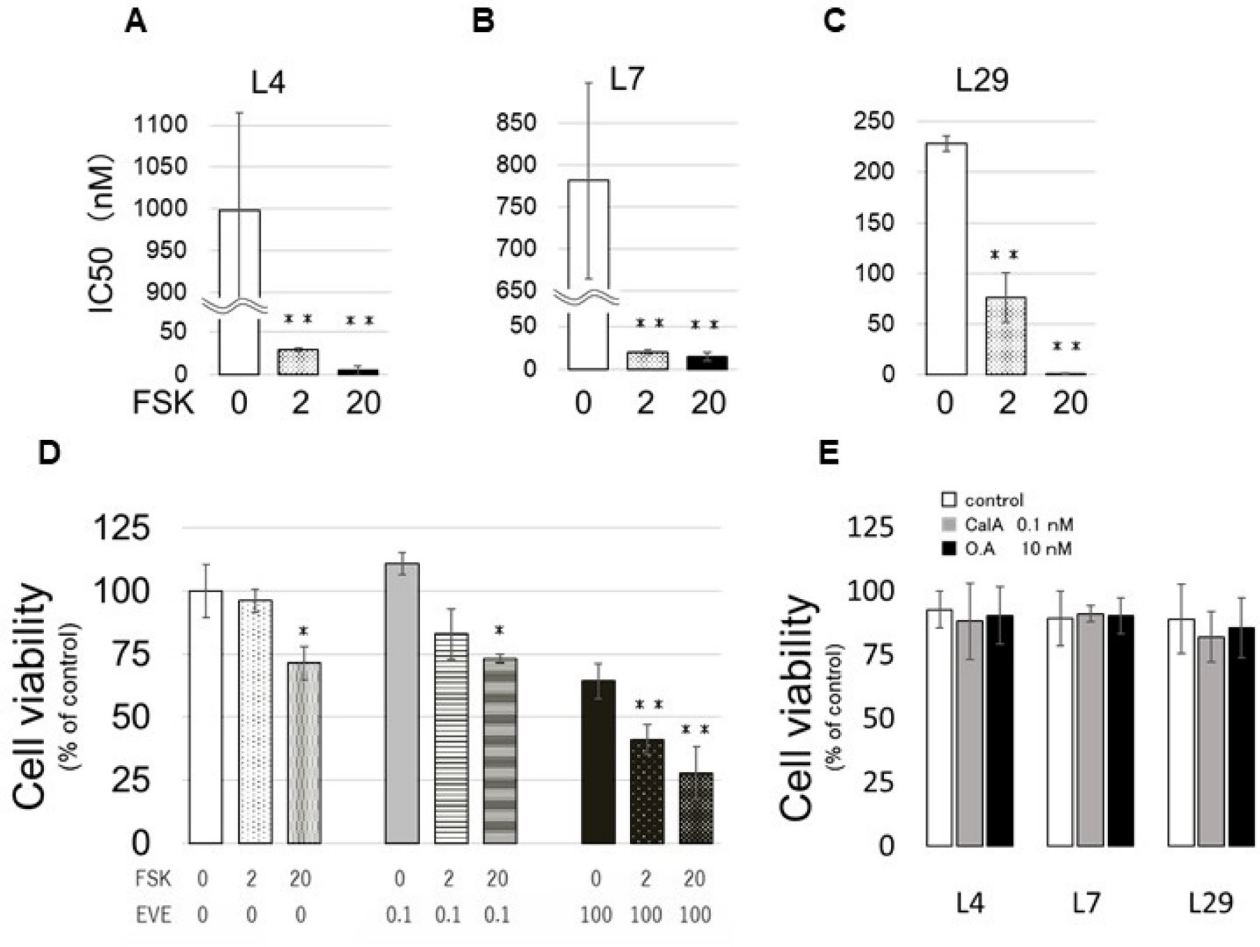
Effect of FSK on EVE sensitivity (**A–C**) EVE-Hi clones, nos. 4 (L4; A), 7 (L7; B) and 29 (L29; C), were incubated with FSK (0, 2 or 20 μM). The medium was changed every 2 days. The number of the cells was measured with a CCK-8 kit. The concentration of EVE associated with a 50% reduction in the cell number indicated the IC50. (**D**) Cells of clone no. 4 cells were counted after incubation with EVE (0.1 or 100 nM) and FSK (0, 2 or 20 μM) for 4 days. (**E**) EVE-Lo cells were incubated with FSK (0 or 20 μM) in medium containing 0.1 nM CalA. The medium was changed every 2 days. The number of the cells was measured with a CCK-8 kit (*n* = 3 per treatment group ^*^P < 0.05; ^**^P < 0.01).

Next, we assessed the IC50 of EVE in the EVE-Lo clones after the treatment with the PP2A activator forskolin (FSK) at two concentrations (2 and 20 μM) and found that FSK treatment was associated with a decrease in IC50 in all clones analyzed (Figure 5A–5C). Clone no. 4 showed the most pronounced decrease: IC50 was about 1000 nM without FSK, 29 nM in the presence of 2 μM FSK, and 5.8 nM in the presence of 20 μM. Additionally, clone no. 4 cells cultivated with EVE (0.1 or 100 nM) and FSK (2 or 20 μM) showed a significant difference in cell viability (Figure 5D). Similar results were obtained with the clones EVE-Lo nos. 7 and 29 (Supplementary Figure 2A and 2B). Finally, to confirm that the effect of FSK was actuated through PP2A, we treated the cells with both FSK and the PP2A inhibitors CalA and OA (Figure 5E). We did not find any significant difference in the viability of cells grown in regular medium or in medium containing the two drugs, confirming that the effect of FSK was due to activation of PP2A.

## DISCUSSION

AI resistance is one of the biggest problems in the treatment of patients with ERα-positive breast cancer. The mTOR pathway plays an important role in cell cycle progression and proliferation and EVE, an mTOR inhibitor, seems to be highly effective in improving AI resistance [12]. For example, the BOLERO-2 clinical trial has examined the combined effect of EVE and an AI in patients with AI-resistant breast cancer and found that treatment with the two drugs is associated with a prolongation of the progression-free survival, confirming the benefits of this treatment for many patients [12, 15]. Unfortunately, approximately 10-15% of the patients were already EVE-resistant at the beginning of the treatment [12]. To investigate the cause of the loss of the therapeutic effect of EVE we established a cellular model of AI-resistant breast cancer. Specifically, we generated 30 clones from MCF-7 cells grown in the absence of estrogen (LTED MCF-7) and investigated their susceptibility and the resistance to EVE.

In this study, we showed that PP2A may be involved in AI resistance. Since the phosphorylation of ERα at S167 depends on the Akt signal pathway and PP2A is a modulator of Akt, this finding is a very reasonable result, which supports our previous report [20].

We did not find any correlation between the sensitivity of the cells to EVE and PP2A expression. Contrarily, high sensitivity to EVE treatment seems to be associated with a reduction in the protein levels of CIP2A, an inhibitor of PP2A. It has been shown that, in triple negative breast cancer, CIP2A has a half-life of 60 hours or more [41]; however, 55% of the newly synthesized CIP2A is degraded in a relatively short time (24 hours). Additionally, CIP2A is targeted to proteasome degradation after translation and Akt activation negatively interferes with CIP2A degradation [42].

The stability of CIP2A is influenced by PP2A [43]. We found that the levels of PP2A in the EVE-Hi and EVE-Lo cells did not significantly change upon EVE treatment. Therefore, there may be other factors that influence the half-life of CIP2A in our model system.

Our knockdown experiments demonstrated that CIP2A is one of the factors playing a role in mediating the sensitivity to EVE. Knockdown of CIP2A was an effective way to improve EVE sensitivity and might be useful to develop novel cancer treatments. In the future, we plan to investigate the molecular mechanism through which CIP2A mediates EVE sensitivity. On the other hand, FSK, a PP2A activator sold as the active ingredient of supplements in Japan, is another drug that might have clinical application for this purpose. Interestingly, some studies have suggested that FSK may be effective also for treating acute leukemia [44, 45] and prostate cancer [46].

CIP2A and PP2A are expected to be very effective therapeutic targets in AI-resistant breast cancers. We believe this study helps understanding if the combined treatment with EVE and FSK might indeed become a novel and effective treatment option for patients with AI resistance.

## MATERIALS AND METHODS

### Cell culture

MCF-7 and T47D cells (human ERα-positive breast cancer cells) were obtained from the American Type Culture Collection (Rockville, MD, USA). MCF-7 and T47D cells were maintained in RPMI 1640 medium (GIBCO BRL, Grand Island, NY, USA) supplemented with 10% fetal bovine serum (FBS) and 1% penicillin/streptomycin at 37° C in a 5% CO_2_-humidified atmosphere incubator. Cells treated with 17 beta-estradiol (E_2_) were cultured in phenol red-free RPMI 1640 medium supplemented with 10% dextran-coated charcoal (DCC)-treated fetal bovine serum (Nichirei Biosciences Inc., Tokyo, Japan) and 1% penicillin/streptomycin. LTED cells, which we used as a model of AI-resistant cells, were derived from a parental cell line by long-term culture in the presence of RPMI 1640 medium containing 10% DCC serum, as described previously [47–49]. For CIP2A knockdown, triplex small interfering RNAs (siRNAs) for CIP2A (Stealth select RNAi) and their control were purchased from Invitrogen (Carlsbad, CA, USA).

### Cell proliferation assay

The viability of cultured cells was determined using a CCK-8 (Dojindo Molecular Technologies, MD, USA) according to the manufacturer's instructions. Cell viability assays were used to compare the effects of EVE, FSK and CalA (Wako Pure Chemical Industries, Ltd., Osaka, Japan) on LTED cells. Briefly, cells (1 × 10^3^ cells/well) were seeded into 96-well plates and grown in medium supplemented with one or more drugs, as indicated, in triplicate wells. Cells were cultured at 37° C in a 5% CO_2_ incubator for 96 hours. The concentrations of EVE used were 0.01, 0.1, 1, 10 and 100 nM; the concentrations of CalA used were 0.01, 0.1 and 1 nM; the concentrations of FSK used were 2 and 20 nM and the concentration of AZD (Cayman Chemical, Ann Arbor, Michigan, USA) was 100 nM. The CCK-8 reagent was added to each well, and the cells are incubated at 37° C in a 5% CO_2_ incubator with saturated humidity for one hour.

### Western blot

Whole-cell lysates were prepared using lysis buffer containing 62.5 mM Tris HCl pH 6.8, 5% 2-mercaptoethanol, 2% sodium dodecyl sulfate [SDS], 5% sucrose, 0.01% Bromophenol Blue (Wako Pure Chemical Industries, Ltd.). The protein content was determined using a RC DC™ Protein Assay kit (Bio-Rad Laboratories, Inc., Hercules, CA, USA) with bovine serum albumin (Sigma-Aldrich Darmstadt, Hamburg, Germany) as standard. For western blot analysis, the samples (5 μg of protein/lane) were separated by 10% SDS-polyacrylamide gel electrophoresis (PAGE) and transferred to a polyvinylidene difluoride (PVDF) membrane (GE Healthcare, Piscataway, NJ, USA). The membrane was pre-incubated with ImmunoBlock (DS Pharma Biomedical Co., Ltd. Osaka, Japan) at 20–25° C for 30 min, and then incubated at 4° C overnight with a primary antibody. The membrane was subsequently washed with TBS-T buffer (20 mM Tris-HCl [pH 7.5], 150 mM NaCl, 0.5% Tween-20) and incubated with a horseradish peroxidase (HRP)-labeled secondary anti-rabbit (1:10000, Bio-Rad Laboratories, Inc., Hercules, CA, USA) or anti-mouse (1:5000, MBL, Nagoya, Japan) antibody for one hour. After the membrane was washed with TBS-T buffer, immunoreactive bands were visualized using the Immobilon Western Chemiluminescent HRP Substrate (Millipore, Billerica, MA, USA). The intensity of the chemiluminescence of specific bands was digitized using the Cool Saver software (ATTO, Tokyo, Japan) and quantified. Rabbit polyclonal antibodies against, ERα (1:2000), pER S167 (1:500), and GAPDH (1:2000) were purchased from Santa Cruz Biotechnology (Santa Cruz, CA, USA). Rabbit poly and monoclonal antibodies against Akt (1:2000), phosphorylated Akt Ser473 (1:1000), S6K (1:1000), phosphorylated S6K (1:1000) and CIP2A (1:1000) were purchased from Cell Signaling Technology, Inc., (Danvers, MA, USA). Anti-PP2A alpha + beta antibody (1:5000) was purchased from Abcam (Cambridge, MA, USA) All antibodies were diluted in the Can Get Signal^®^ Immunoreaction Enhancer Solution (Toyobo, Inc., Osaka, Japan).

### RNA extraction and quantitative PCR

Total RNA was extracted from treated cells using the TRIzol reagent (Qiagen, Hilden, Germany) and reverse transcribed using the PrimeScript RT reagent kit (TaKaRa Sake USA, Torrance, CA, USA). Quantitative PCR (qPCR) was performed in triplicate using the ABI Perkin-Elmer Prism 7300HT Sequence detection system (Applied Biosystems, Foster City, Ca, USA). Taqman gene expression assays (Applied Biosystems) were used to detect expression of CIP2A (Taqman Accession ID Hs00405413_m1) and PP2A (Hs00988483_m1); GAPDH (Hs999999905_m1) was used as a housekeeping gene. Relative quantities were determined using the ΔΔCt method, according to the manufacturer›s instructions.

### Statistics

All experimental data comparing more than two groups were analyzed by one-way ANOVA followed by Fisher's protected least significant difference (PLSD) test. When differences were significant, subsequent analysis with the Post Hoc test with the Bonferroni correction was performed. Other statistical comparisons were conducted by a two-tailed unpaired *t*-test. Data are represented as the mean ± the standard error of the mean (SEM). Data were considered significant when *P* < 0.05.

## SUPPLEMENTARY MATERIALS AND FIGURES



## Abbreviations

AI: Aromatase inhibitor
AZD: AZD5363
CalA: calyculin A
CCK-8: cell counting kit 8
CIP2A: cancerous inhibitor of PP2A
DCC: dextran-coated charcoal
E_2_: estradiol
ERα: estrogen receptor alpha
EVE: everolimus
FBS: fetal calf serum
FDA: U. S. Food and Drug Administration
FSK: forskolin
GAPDH: glyceraldehyde 3-phosphate dehydrogenase
HER2: human epidermal growth factor receptor 2
HRP: horseradish peroxidase
LTED: long-term estrogen-deprived
no(s).: number(s)
mTOR: mammalian target of rapamycin
OA: okadaic acid
pER: phosphorylation of ERα
PI3K: phosphatidylinositol 3-kinase
PLSD: protected least significant difference
PP2A: protein phosphatase type 2A
PVDF: polyvinylidene difluoride
qPCR: quantitative PCR
S: serine
SDS-PAGE: sodium dodecyl sulfate-polyacrylamide gel electrophoresis
SEM: standard error of the mean
siRNAs: small interfering RNAs
