# Supplementary material for: Forskolin increases the effect of everolimus on aromatase inhibitor-resistant breast cancer cells
Source: Oncotarget. 2018 May 4;9(34):23451–61. doi: 10.18632/oncotarget.25217 (PMC5955115; doi:10.18632/oncotarget.25217)
Supplement: Supplementary file 1 [file oncotarget-09-23451-s001.pdf]

Forskolin increases the effect of everolimus on aromatase inhibitor-resistant breast cancer cells

SUPPLEMENTARY MATERIALS

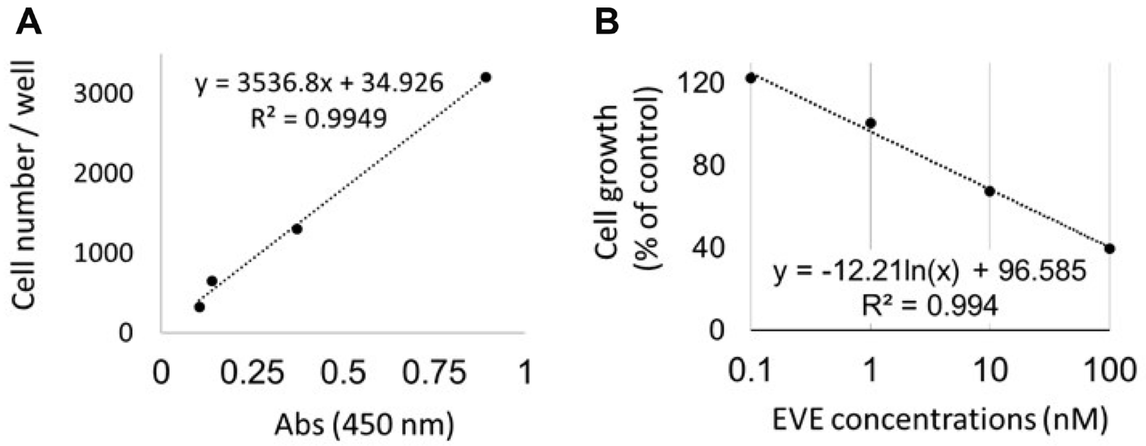

Supplementary Figure 1: Procedure to measure EVE IC50. (**A**) Calibration curve to calculate the number of cells per well. (**B**) Calibration curve to calculate EVE IC50 from the cell number.

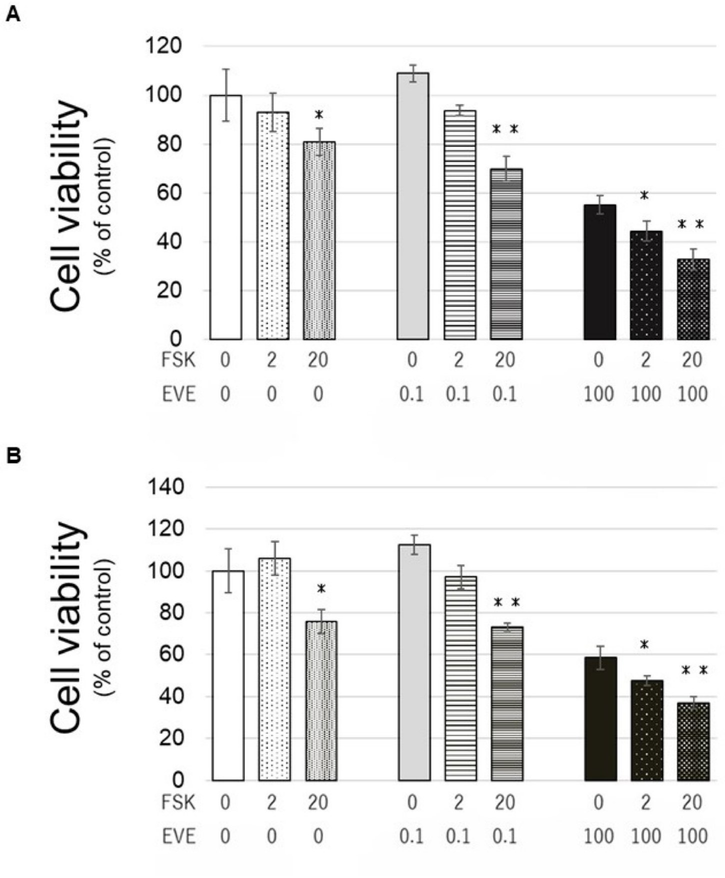

Supplementary Figure 2: Effect of FSK on EVE sensitivity in clones nos. 7 and 29. (**A–B**) Cells of clone no. 7 (A) and no. 29 (B) were counted after incubation with EVE (0.1 or 100 nM) and FSK (0, 2 or 20 µM) for 4 days. The medium was changed every 2 days. The number of the cells was measured with a CCK-8 kit (n = 3 per treatment group $^{*}P < 0.05$; $^{**}P < 0.01$) The concentration of EVE associated with a 50% reduction in the cell number indicated the IC50.
